# Alcohol use among high school learners in rural areas of Limpopo province

**DOI:** 10.4102/sajpsychiatry.v25i0.1183

**Published:** 2019-10-17

**Authors:** Olivia M. Maserumule, Linda Skaal, Sello L. Sithole

**Affiliations:** 1 Department of Public Health, University of Limpopo, Polokwane, South Africa; 2 School of Social Sciences, University of Limpopo, Polokwane, South Africa

**Keywords:** alcohol abuse, audit, alcohol dependence, highs, rural areas, high school learners, drinking behaviour

## Abstract

**Background:**

Alcohol use is a serious public health concern among youth in South Africa and worldwide.

**Aim:**

To determine the factors contributing to alcohol use among high school learners in the rural areas of Limpopo province.

**Setting:**

The Greater Marble-Hall municipality, Sekhukhune district in Limpopo province.

**Method:**

A quantitative, cross-sectional study design was conducted on 314 learners from three high schools in a rural area in Limpopo. A stratified random sampling technique was used to select learners from 11 to 25 years of age. The drinking behaviour was classified according to predetermined Alcohol Use Disorders Identification Test (AUDIT). Data were analysed using SPSS Software v23.0.

**Results:**

More than half of the respondents were consuming alcohol 169 (53.8%). Also, 173 (55.1%) of respondents had parents who consume alcohol and 204 (65%) had friends who drank alcohol. Most respondents were classified as low-risk drinkers (AUDIT score < 8) and a quarter of the respondents were classified as almost dependent on alcohol (AUDIT score > 13). Significant associations were found between learners’ alcohol consumption and parents and friends who drank alcohol (*p* = 0.000; *p* = 0.000, respectively).

**Conclusion:**

Alcohol use was prevalent among high school learners in the area under investigation. Also, learners who had parents and friends who consume alcohol were more likely to consume alcohol. Further, learners who were classified as almost dependent on alcohol needed urgent intervention as their health-related quality of life was likely to be poor.

## Introduction

Alcohol use among young and old people is one of the most significant public health and social problems worldwide.^1,2^ Despite the adverse effects of excessive alcohol consumption on health, alcohol beverages are consumed throughout the world. Moreover, alcohol consumption is rated as the world’s third largest risk factor for diseases.^3^ The harmful use of alcohol is a causal factor of more than 60 major types of diseases and injuries that include Alcohol Use Disorders (AUD), cancer, cardiovascular diseases, foetal alcohol syndrome, diabetes mellitus, suicide and violence to name a few.^4^ Worldwide, annual alcohol consumption was equal to 6.13 litres of pure alcohol consumed by persons aged 15 or older in 2005.^4^ The 2018 World Health Organization (WHO) Global Status Report on Alcohol Consumption^4^ in 189 countries places South Africa as the 19th highest consumer of alcohol. Currently, South Africa consumes 11.5 litres of pure alcohol and it is estimated that this will increase to 12.1 litres by 2025.^4^ to the National Council on Alcoholism and Drug Dependence,^3^ 23% of youth aged 12 to 20 consume alcohol. Heavy episodic drinking is one of the most important indicators for acute consequences of alcohol use and it is high in countries such as Brazil and South Africa.^5^ Thus, 4% of all deaths worldwide are attributed to excessive alcohol consumption.^4^ Furthermore, WHO Global Status Report on Alcohol and Health^5^ states that globally, age, gender, familial risk factors, socioeconomic factors, culture and alcohol controlled and regulations are factors contributing to alcohol consumption among youth. Globally, men have been reported to be more likely to consume more alcohol than women.^6^

World Health Organization^4^ highlights that South Africans consume 10 litres of pure alcohol in a year which is much higher than the worldwide consumption.^4^ Furthermore, Makhubele^7^ posits that this figure is likely to be higher if home-made alcohol is included, making South Africa the heavy drinking country in Africa.^8^ According to the South African Department of Education, alcohol consumption is estimated at 28% of which 7% is classified as high risk as determined by the Alcohol Use Disorders Identification Test (AUDIT) scale.^9^ In South Africa, many high school learners have reported that they have tried drinking alcohol and many of them still drink at regular intervals.^10^ Of concern is that alcohol initiation age has reduced significantly, about 12% of youth in South Africa start drinking by the age of 13.^1,11^ Mothibi^12^ conducted a study in rural communities of Limpopo province and found that adolescents started using alcohol between the age of 13 and 14. It is worrisome that despite legislation that prohibits the sale of alcohol to minors, they can acquire alcohol through direct purchase themselves through older friends and family members, or by stealing from parents and other adults who drink alcohol.^13^ Themane^13^ further indicates that accessibility to alcohol to rural school learners is not unique to Limpopo province. Although the legal drinking age of alcohol in South Africa is 18, statistics show an alarming increase in alcohol use with binge drinking being more prevalent among youth younger than 18 years in South Africa.^14^ This can be attributed to the fact that during transition stages from childhood to teen years, biological changes drive young people to try new things in their lives, such as drinking alcohol.^15^ In a study conducted by the Department of Social Development in Southern KwaZulu-Natal, it is reported that 53% of the population started consuming alcohol at a young age.^9^

Limpopo province is largely rural and alcohol (all types including traditionally brewed alcohol) is used mostly in traditional and cultural practices and rituals. This sometimes exposes adolescents to alcohol use at a young age.^10^ Early initiation to alcohol use among adolescents is a cause for concern. Often co-existing with this alcohol use is teenagers engaging in risky sexual behaviours whilst intoxicated; most of whom do so without knowing the HIV status of the partner.^16^ Furthermore, the extent to which alcohol is used in Limpopo is not known; hence, we conducted this study to determine alcohol use among high school learners in rural areas of Limpopo province.

## Methods

### Study method

The study utilised a quantitative approach and a cross-sectional research design. Data were collected in three high schools in Toitskraal, a rural area in Limpopo province. These schools are situated in deep rural areas of the Greater Marble-Hall municipality, Sekhukhune district. These schools cater for learners from low socio-economic backgrounds. Ethical clearance was obtained from the Ethics Committee of the University (TREC:116/2015:PG) and permission to collect data was also obtained from the Department of Education. A written consent letter was signed by parents and a written assent letter was signed by learners.

### The study participants

A total of 314 learners were sampled from a population of 1030 learners from grade eight to grade 12 from all three high schools in Toitskraal. Only learners who were enrolled from grade eight to grade 12 during data collection and learners with parental consent and who assented to participate were included in the study. A stratified random sampling technique was used to select participants from those learners who consented and assented per school and per grade. Thus the population was known in line with Krejicie and Morgan,^17^ a table for determining the sample size was used.

### Data collection

Data were collected using a close-ended questionnaire containing 15 questions over a period of 2 months.

It was developed in English and translated to Sepedi. It included two sections: sociodemographic profile and WHO’s AUDIT. The questionnaire was completed by learners during their free periods, lunch breaks and after school to ensure that there were no interruptions of scheduled programmes. The questionnaire was checked for completeness on submission and no missing data on submitted questionnaires. Reliability of the instrument was ensured through pilot testing because this was a pre-validated tool; its reliability has been reported to be 1.0 which makes it a reliable instrument to assess alcohol use. The pre-validated standard WHO-AUDIT Questionnaire was used.

The AUDIT is a 10-question screening tool developed by the WHO and it is used for detection of risky and high-risk alcohol drinking. Alcohol Use Disorders Identification Test classifies levels of drinking into four categories: mainly low risk, risky, high-risk and almost dependent.

### Data analysis

Data were loaded into and analysed using Statistical Package for the Social Science 23.0 software.

Descriptive and inferential statistical analysis was carried out. From AUDIT scores: a score of less than 8 is associated with low risk drinking, a score of 8 or more indicates harmful or hazardous drinking, a score of 13 or more in women and 15 or more in men indicates alcohol dependence. A Chi-squared test was used to determine associations between variables. The binary logistical regression analysis was used to calculate odds ratios and predictors of alcohol use among adolescents at 95% confidence interval.

## Ethical consideration

The authors declare that they obtained ethical clearance (TREC/116/2015:PG); parental consent and assent prior to data collection.

## Results

Table 1 describes the sociodemographic data of learners who participated in the study. Of the 314 learners, 152 (48.4%) were men and 53.8% were greater than or equal to 17 years of age. Furthermore, 173 (55.1%) of the learners reported that they had parents who drink alcohol and 204 (65%) reported that they had friends who drink alcohol. On self-drink, 169 (53.8%) of respondents currently drink alcohol and 142 (45.2%) did not drink alcohol.

**TABLE 1:** Sociodemographic profile of learners (*N* = 314).

Variables	Frequency	Percentage
**Gender**		
Men	152	48.4
Women	162	51.6
**Age†,‡**		
11–14 years	53	16.9
15–16 years	92	29.3
17 and more	169	53.8
**Do you currently drink alcohol?**		
Yes	169	53.8
No	142	45.2
**Parents who drink**		
Yes	173	55.1
No	141	44.9
**Friends who drink**		
Yes	204	65.3
No	109	34.7

† , mean = 16.8.

‡ , standard deviation = 2.28.

**FIGURE 1:**
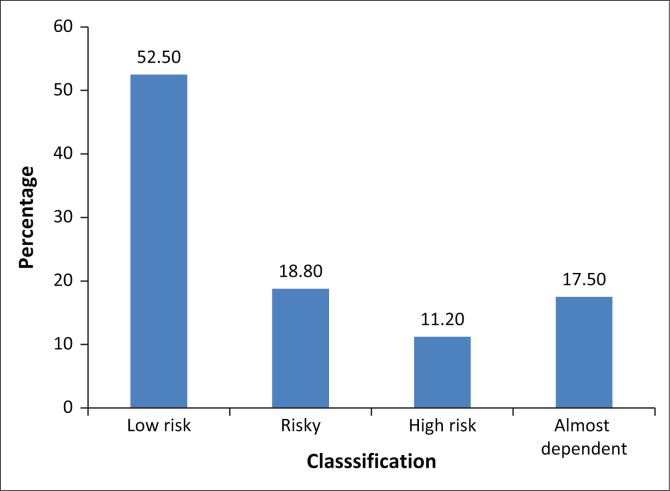
Alcohol Use Disorders Identification Test classification of alcohol use by respondents (*N* = 314).

Figure 1 shows that most respondents 164 (52.5%) were low-risk drinkers (AUDIT scores < 8) followed by 59 (18.8%) who were risky drinkers (AUDIT scores 8–12) and 54 (17.5%) were almost dependent drinkers (AUDIT scores > 12).

Table 2 shows alcohol consumption in terms of socio-demographic profile. There was no significant association between genders and age of the respondents and AUDIT classification of alcohol drinking (*p* = 0.535). In terms of religion, there was a significant association (*p* = 0.001). There was a significant association between alcohol consumption; parents and friends who drink alcohol (*p* = 0.001; *p* = 0.001). This means that having parents who drink alcohol is likely to influence an individual to follow the pattern.

**TABLE 2:** Alcohol Use Disorders Identification Test classification according to Sociodemographic profile.

SDP variables (*N* = 314)	Low risk %	Risky %	High risk %	Almost dependent %	*x*^2^	*p*
**Gender**						
Male (*N* = 152)	51.9	19.7	13.1	15.1	2.185	0.535
Female (*N* = 162)	53.0	17.9	19.2	19.7		
**Age group**						
11–14 (*N* = 53)	64.1	16.9	15.0	3.7	10.544	0.104
15–16 (*N* = 92)	46.7	21.7	9.7	21.7		
≥ 17 (*N* = 169)	52.0	17.7	10.6	19.5		
**Religion**						
Christian (*N* = 279)	53.4	18.6	10.0	17.9	21.533	0.001
Islamic (*N* = 12)	91.6	0.0	0.0	8.3		
Other (*N* = 23)	21.7	30.4	30.4	17.3		
**Parents who drink**						
Yes (*N* = 173)	34.1	27.1	16.7	22.1	54.589	0.000*
No (*N* = 173)	75.1	8.5	4.2	12.0		
**Friends who drink**						
Yes (*N* = 109)	92.6	0.9	3.6	3.2	112.670	0.000*
No (*N* = 204)	31.3	28.4	15.1	25.0		

* , Statistical significance at 95% CI; *x*^2^, Chi Square.

Table 3 shows that parents who drink and friends who drink were significant predictors of drinking behaviours of learners and not gender (OR = 1.042, 2.321; *p* = 0.000; 0.000), respectively.

**TABLE 3:** Binary logistic regression analysis between learners who drink, gender, parents and friends who drink alcohol.

Variables in the equation	B	s.e.	Wald	df	Sig.	Exp (B)
**Step 1**†						
Parents drink	1.047	0.287	13.327	1	0.000	2.848
Friends drink	2.321	0.318	53.337	1	0.000	10.184
Gender	−0.287	0.280	1.055	1	0.304	0.750
Constant	−2.018	0.583	11.993	1	0.001	0.133

B, coefficient; s.e., standard error; Wald, Wald test; df, degrees of freedom; Sig., significance; EXP (B), exponentiation of the B coefficient.

† , Variable(s) entered on Step 1: parents drink, friends drink and gender.

## Discussion

Our results revealed that the majority of learners were between 17 and 25 years of age implying that they are at a stage characterised by many social, emotional and physical pressures.^9^ Furthermore, the social cognitive theory posits that an individual’s knowledge acquisition can be directly related to observing others within the context of social interactions, experiences and outside media influences.^18^ As one grows, the transition to adulthood can take place in different forms and over a wide range of ages from the teens through the mid to late 20s.^19^ Moreover, young adulthood is a stage of life marked by change and exploration, whereby teenagers begin to make their own decisions and sometimes engage in unhealthy behaviours such as drinking alcohol.^20^ Studies reported young adults tend to be influenced by friends at this stage because of a need to belong to a certain group; hence, parents tend to have a less direct influence on their drinking behaviour, which is mostly performed in secrecy.^21,22^ This finding also implies that the learners in this rural area are likely to drink alcohol and engage in other forms of risk-taking behaviours based on their age.

Rural areas are characterised by limited educational and recreational facilities^23^; and it appears that some learners do not explore other means of entertainment except opting for drinking. The taverns close to some of these schools make it easy for learners to access alcohol.^24^ In an article in the City Press, learners whose schools are nearer to taverns had more access to alcohol.^25^ Of concern is the fact that one-third of learners aged ≤ 14 years reported that they currently consume alcohol, two of which were classified as almost dependent on alcohol at such a young age. Such patterns of alcohol use among these adolescents is a cause for concern as early initiation of alcohol drinking is associated with a range of negative consequences, including unprotected sexual behaviour and risk of sexually transmitted infectious diseases such as STIs and HIV and risk of unwanted pregnancies.^26^ It is very difficult to immediately diagnose some adolescents as problem drinkers because most of them start their drinking in secrecy. National Institute on Alcohol Abuse and Alcoholism (NIAAA)^27^ reported that most people find it difficult to distinguish when normal drinking becomes problem drinking and it further emphasised that screening for alcohol use is of importance in order to identify those at risk of becoming problem drinkers.^27^

Studies have consistently shown that men consume more alcohol and have more alcohol-related problems than women.^2,20^ In the KwaZulu-Natal, Western Cape and Gauteng provinces of South Africa, studies found that more men than women consumed and binged on alcohol.^9,20,28,29^ Although there was no statistical significance between gender and AUDIT classification, it is of concern that slightly more number of girls were found to be classified as almost dependent on alcohol rather than boys in our study. This finding is in contrast with a study conducted by Onya et al.^26^ in the Limpopo province where gender was found to be a significant predictor of alcohol use with men having double the odds of ever using alcohol than women.^26^ This was primarily because of easy access to alcohol and perceptions that there is nothing wrong with adolescent boys who drink.^26^ One should note that this study by Onya et al.^26^ was conducted more than 7 years ago and new developments show no statistical variations in drinking behaviours in relation with gender as evidenced by our study and others also found that when it comes to drinking, the gender gap is disappearing as girls are now surpassing boys.^30,31^

Evidence suggests that the use of alcohol constitutes one of the most risk-taking forms of behaviour among adolescents in secondary schools.^13,32^ Although most learners were non-problem drinkers, they are susceptible to alcohol use because they are at vulnerable ages where peer influence can play a significant role in shaping behaviours. According to the Prevention of and Treatment for Substance Abuse Act, primary prevention measures delay the onset of alcohol use through risk reduction by altering behaviours or exposures that can lead to alcohol use. It is applicable to learners who have not yet been initiated into alcohol and drug use.^33^ Furthermore, primary prevention measures can be directed towards health promotion activities involving educational and psycho-social interventions.^34^ Learners who were found to be risky drinkers and those who were almost dependent on alcohol require urgent intervention, seeing that they are at risk of not finishing school, adding to a vicious cycle of poverty.^35^ It can, therefore, be deduced that alcohol dependence is emerging as a threat among rural learners and it is clear that social factors such as parents and peer drinking behaviours influence alcohol use, as found in this study and others.

Factors that contribute to adolescent drinking were also investigated in this study. We found that more than two-thirds of the respondents have friends who drink alcohol. This means that having friends who drink alcohol significantly increases the likelihood of an individual to drink alcohol as well.^24^ Peers have been known to be a factor that influences behaviour among youth because they are going through a period of change in emotional attachment as youth strive to become more autonomous and the attachment extends beyond parents to peers.^36^ Studies conducted in South Africa by Mohasoa and Bendsen also found that adolescents depended on their peers for emotional care and support and they have no choice but to give in to everything that their peers offered.^21,37^ Adolescents would drink alcohol to please friends and to gain a sense of belonging. There seems to be a consensus in studies that they drink to please peers; this was also found in our study.^21,24,36,37^ Interestingly, the parental factor was also found to be significantly influencing adolescent drinking in our study. Chauke et al.^1^ reported that parents who use alcohol are likely to predetermine the future alcohol involvement for their children. It can be inferred that both peer and parent factors influence drinking habits of adolescents in our study.

## Limitations of the study

The population in this study comprised high school learners from three schools in one village of Limpopo province. This has implications for the generalisability of the findings for the province. The self-reported data are subject to bias as respondents could have over or under-reported their rate of alcohol use.

## Conclusion

This study revealed that over half of learners drink alcohol and they were risky drinkers; a few were almost dependent on alcohol. We conclude that alcohol use is highly prevalent among high school learners and alcohol dependence is becoming a threat at a very early age in this rural area of the Limpopo province. Peer and parental factors were also found to be significant predictors of alcohol use among adolescents in this study.

### Recommendations

We, therefore, recommend that interventions to prevent and reduce alcohol use and all factors influencing alcohol use among adolescents be investigated to inform any type of intervention. The Department of Education should intensify the implementation of school-based alcohol prevention programmes. This drive should also include community mobilisation to assist in regulating the opening and closing hours of taverns nearer to schools.
